# Supplementary material for: Bone mineral density among virologically suppressed Asians older than 50 years old living with and without HIV: A cross-sectional study
Source: PLoS One. 2022 Nov 21;17(11):e0277231. doi: 10.1371/journal.pone.0277231 (PMC9678298; doi:10.1371/journal.pone.0277231)
Supplement: Attachment — Submitted filename: PONE-D-22-09858_Rec.pdf [file pone.0277231.s001.pdf]

The present manuscript evaluated BMD in virologically suppressed men and women living with HIV in an elderly Asian population. The authors found low BMD in men and women with HIV however BMD did not differ after controlling for age, BMI, and other traditional osteoporotic risk factors. The following findings add to a body of literature and would be beneficial for those managing the treatment of HIV, specifically among the Asian population. However, the authors should consider the following recommendation before submitting it for publication.

1. Improve mechanics, table organization, spacing as well as formatting of tables in the document.
2. A few lines, for example, Line 57 appear to need citations.
3. The reproductive history in Table 1 is unclear. Is menstruation exhausted referring to the number of women that have reached menopause?
4. Empty boxes in tables 3 and 4
5. What units are being presented in table 6?
6. Were any of the women in the study on hormonal replacement therapy?
7. In the abstract it is written, “After adjusting for age, BMI, 45 and other traditional osteoporotic risk factors, BMD of virologically suppressed older PWH did not differ from participants without HIV”, however, no data is shown highlighting adjusted BMD. A similar statement is made in the discussion, lines 259-260, which states, “after adjusting for clinical risk factors listed above, there was no difference in BMD at any sites between PWH and people living without HIV”, however, adjusted BMD values are not in the manuscript. All that is shown in table 2, which has the unadjusted BMD values, which are lower in people with HIV compared to people without HIV.
8. Elevated circulating levels of bone turnover markers such as P1NP and Ctx can be indicative of dysregulated bone turnover, which can also drive increased fracture risk. It is worth discussing what this could mean for long-term bone quality for people living with HIV. For instance, it has been shown that there is reduced bone microarchitecture in people with HIV despite comparable BMD relative to HIV age-matched sex-matched HIV negative persons, therefore even if there are no significant BMD differences there are other factors outside of BMD that may drive fracture risk.
