# Supplementary material for: Bone mineral density among virologically suppressed Asians older than 50 years old living with and without HIV: A cross-sectional study
Source: PLoS One. 2022 Nov 21;17(11):e0277231. doi: 10.1371/journal.pone.0277231 (PMC9678298; doi:10.1371/journal.pone.0277231)
Supplement: Attachment — Submitted filename: Response to Reviewers.docx [file pone.0277231.s002.docx]

**Reviewers' comments:**

**Reviewer #1:**

1. Improve mechanics, table organization, spacing as well as formatting of tables in the document.

RESPONSE: Thank you for the suggestion. We have managed to make sure that all the tables are well formatted and easy to read.

2. A few lines, for example, Line 57 appear to need citations.

**RESPONSE:** Thank you for pointing out this this. We have carefully rechecked to ensure that all the sentences had proper citations, including the sentence in line 58-59. Reference 3, 4, and 50 were added into the References section.

3. The reproductive history in Table 1 is unclear. Is menstruation exhausted referring to the number of women that have reached menopause?

RESPONSE: Thank you for. Yes, “menstruation exhausted” referred to the number of women that have reached menopause. We have added “n (%)” after each category of reproductive history in Table 1.

4. Empty boxes in tables 3 and 4

RESPONSE: All variables with p-values ≤ 0.20 from the univariate analyses were included in the multivariable regression model. Therefore, all the empty boxes in Table 3 and Table 4 were left blank because these variables that were not significant in the univariate models were not included in the multivariate analyses. The detailed methodology regarding the adjusted models was provided in the Statistical analysis section of, line 132-133.

5. What units are being presented in table 6?

RESPONSE: We have added the units of P1NP, CTX and 25(OH)D in Table 6.

6. Were any of the women in the study on hormonal replacement therapy?

RESPONSE: All the women in the study were not on hormonal replacement therapy.

7. In the abstract it is written, “After adjusting for age, BMI, 45 and other traditional osteoporotic risk factors, BMD of virologically suppressed older PWH did not differ from participants without HIV”, however, no data is shown highlighting adjusted BMD. A similar statement is made in the discussion, lines 259-260, which states, “after adjusting for clinical risk factors listed above, there was no difference in BMD at any sites between PWH and people living without HIV”, however, adjusted BMD values are not in the manuscript. All that is shown in table 2, which has the unadjusted BMD values, which are lower in people with HIV compared to people without HIV.

RESPONSE: Analysis of the comparison of adjusted BMD between HIV+ and HIV- participants of both sexes (shown below) indicated that the BMD values after adjustment were comparable between HIV- and HIV+ of both sexes.

*Comparison of adjusted BMD between HIV+ and HIV- participants of both sexes*

| *Site* | *HIV status* | *Absolute BMD (g/cm^2^)* | *Adjusted model** | | *P-value^ⱡ^* |
| --- | --- | --- | --- | --- | --- |
|  |  |  | *Mean difference* | *95% CI* |  |
| *Men (n=297)* | | | | | |
| *Lumbar spine* | *uninfected* | *0.96 (0.14)* | *ref* | *-* |  |
|  | *infected* | *0.94 (0.16)* | *-0.002* | *(-0.04, 0.04)* | *0.93* |
| *Total hip* | *uninfected* | *0.91 (0.13)* | *ref* | *-* |  |
|  | *infected* | *0.86 (0.13)* | *-0.03* | *(-0.06, 0.003)* | *0.076* |
| *Femoral neck* | *uninfected* | *0.74 (0.12)* | *ref* | *-* |  |
|  | *infected* | *0.71 (0.11)* | *-0.02* | *(-0.04, 0.01)* | *0.26* |
| *Women (n=184)* | | | | | |
| *Lumbar spine* | *uninfected* | *0.88 (0.18)* | *ref* | *-* |  |
|  | *infected* | *0.85 (0.16)* | *-0.04* | *(-0.08, 0.01)* | *0.12* |
| *Total hip* | *uninfected* | *0.81 (0.17)* | *ref* | *-* |  |
|  | *infected* | *0.79 (0.13)* | *-0.01* | *(-0.05, 0.03)* | *0.56* |
| *Femoral neck* | *uninfected* | *0.66 (0.21)* | *ref* | *-* |  |
|  | *infected* | *0.64 (0.12)* | *-0.02* | *(-0.06, 0.02)* | *0.32* |

**Adjusted for age, BMI, and smoking status in men and for age, BMI, and menstruation
 status in women; ^ⱡ^ P-values were estimated by linear regression models*

- We have demonstrated in Table 3 and Table 4 that HIV status was not correlated with BMD in both men and women in the multivariate analyses. So, we did not include the table above in our manuscript.

However, we have provided p-value “P>0.1” in the abstract as the followings:

*“After adjusting for age, BMI, 45 and other traditional osteoporotic risk factors, BMD of virologically suppressed older PWH did not differ from participants without HIV (P>0.1)”.*

- In Discussion section, we have also added the table references “(Table 3 and Table 4)” in line 271-273 as the following**:**

*“…after adjusting for clinical risk factors listed above, there was no difference in BMD at any sites between PWH and people living without HIV* (Table 3 and Table 4).”

8. Elevated circulating levels of bone turnover markers such as P1NP and CTX can be indicative of dysregulated bone turnover, which can also drive increased fracture risk. It is worth discussing what this could mean for long-term bone quality for people living with HIV. For instance, it has been shown that there is reduced bone microarchitecture in people with HIV despite comparable BMD relative to HIV age-matched sex-matched HIV negative persons, therefore even if there are no significant BMD differences there are other factors outside of BMD that may drive fracture risk.

RESPONSE: We have expanded our discussion on page 21 in Discussion section, line 335-337, as the followings:

*“Fifth, index of bone microarchitectural quality, such as trabecular bone score, was not assessed in our study. It has been shown that PWH had worse bone microarchitecture than age- and sex-matched HIV negative persons [50]. Future studies evaluating the bone quality together with BMD may improves fracture prediction in older PWH with virological suppression. Finally, this study is a cross-sectional design, while a longitudinal study would have provided more information on the BMD changes over time and the fracture incidence, as well as the factors that influence bone loss and fracture.”*

**Reviewer #2:**

1. Ideally, a longitudinal design would have been used. The authors should comment on the rationale for only presenting cross sectional data for this cohort which is in long term follow up.

RESPONSE: Thank you for your suggestion. Currently, the longitudinal study of these participants is undergoing to follow-up on measuring the changes in BMD and the fracture incidence, as well as identifying the factors that influence bone loss and fracture.

- In the current study, we report the outcome of BMD, calciotropic hormones, vitamin D status, and bone turnover markers at the patients’ baseline visit compared to HIV-uninfected controls.
- We have expanded our discussion on page 22 in Discussion section, line 339-341.

2. The reason that 38% of those on TDF ART were on a half dose should be further detailed.

RESPONSE: All treatment regimens and doses were based on the physician’s decision since this is an observational study, and the reason behind it may probably due to the reduced creatinine clearance or low body weight of patients.  We have added more detail in Results section (line 160-162) as the following:

*“Among these, 35% used TDF at half dose. Of note, since this is an observational study, all ART regimens and doses were based on the physician’s decision and local guidelines, and the reason behind it may probably because of reduced creatinine clearance or low body weight of patients.”*
